# SARTAB, a scalable system for automated real-time behavior detection based on animal tracking and Region Of Interest analysis: validation on fish courtship behavior

**DOI:** 10.3389/fnbeh.2024.1509369

**Published:** 2024-12-05

**Authors:** Tucker J. Lancaster, Kathryn N. Leatherbury, Kseniia Shilova, Jeffrey T. Streelman, Patrick T. McGrath

**Affiliations:** ^1^McGrath Lab, Georgia Institute of Technology, School of Biological Sciences, Atlanta, GA, United States; ^2^Streelman Lab, Georgia Institute of Technology, School of Biological Sciences, Atlanta, GA, United States

**Keywords:** behavior, computational ethology, cichlid fish, Computer Vision, Machine Learning, real-time analysis

## Abstract

Methods from Machine Learning (ML) and Computer Vision (CV) have proven powerful tools for quickly and accurately analyzing behavioral recordings. The computational complexity of these techniques, however, often precludes applications that require real-time analysis: for example, experiments where a stimulus must be applied in response to a particular behavior or samples must be collected soon after the behavior occurs. Here, we describe SARTAB (Scalable Automated Real-Time Analysis of Behavior), a system that achieves automated real-time behavior detection by continuously monitoring animal positions relative to behaviorally relevant Regions Of Interest (ROIs). We then show how we used this system to detect infrequent courtship behaviors in *Pseudotropheus demasoni* (a species of Lake Malawi African cichlid fish) to collect neural tissue samples from actively behaving individuals for multiomic profiling at single nucleus resolution. Within this experimental context, we achieve high ROI and animal detection accuracies (*mAP@*[.5 : .95] of 0.969 and 0.718, respectively) and 100% classification accuracy on a set of 32 manually selected behavioral clips. SARTAB is unique in that all analysis runs on low-cost, edge-deployed hardware, making it a highly scalable and energy-efficient solution for real-time experimental feedback. Although our solution was developed specifically to study cichlid courtship behavior, the intrinsic flexibility of neural network analysis ensures that our approach can be adapted to novel species, behaviors, and environments.

## 1 Introduction

In recent years, we have seen the emergence of numerous advanced animal behavior analysis tools based on Deep Learning (DL) technologies (Mathis and Mathis, 2020; Pereira et al., 2020; Datta et al., 2019; Couzin and Heins, 2023). These tools have found applications throughout our experimental workflows, driving improvements in both data acquisition (Karashchuk et al., 2021; Bala et al., 2020; Mathis et al., 2022) and interpretation (Luxem et al., 2022; Hsu and Yttri, 2021; Yamazaki et al., 2019). Methods from the field of Computer Vision (CV) have proven particularly useful for extracting behaviorally relevant low-level representations (e.g., positions, poses, and kinematics) directly from high-volume video of freely behaving animals (Dunn et al., 2021) - an increasingly ubiquitous data format (Von Ziegler et al., 2020). For the first time in history, it is possible to capture and efficiently analyze behavioral data at something approaching the true range of spatial, temporal, and sensory scales involved (Gomez-Marin et al., 2014).

The remarkable capabilities of DL-based analysis, however, come at a steep computational cost. This is particularly true for video analysis due to the complexity, volume, and high dimensionality of video data. Most applications therefore focus on offline analysis, in which stored video data are analyzed in bulk. This allows for better sharing of limited GPU (Graphics Processing Unit) resources as well as significantly faster inference due to batch processing efficiencies (NVIDIA, 2015). This offline approach, while practical in many settings, precludes an important class of experimental designs that require continuous real-time analysis. Examples include (1) closed-loop experiments in which a stimulus (such as a reward, a sensory cue, or even direct neurostimulation) must be presented in response to a particular behavior, and (2) human-in-the-loop experiments where samples must be collected soon after the behavior of interest occurs (e.g., when profiling transient behavior-dependent hormonal, transcriptomic, or epigenomic states; Johnson et al., 2020a; Baran and Streelman, 2020). Although such experiments can often be achieved through manual observation of behaving individuals or constrained experimental design, this involves either investing significant person-power to constant manual observation or restricting the natural behavioral repertoire to simplify measurement. DL-based approaches, with their proven track record of achieving human-level accuracy in complex naturalistic environments (Sturman et al., 2020), present an obvious solution – so long as we can achieve real-time performance.

Despite the inherent challenges, examples of real-time DL-based animal behavior analysis do exist. One common approach requires access to a GPU-enabled computer or server, either directly or via the local network, and configuring the DL model to process each frame as it arrives (rather than processing frames in large batches for efficiency, as is common when analyzing stored video). Examples of this approach include DLC-Live! (Kane et al., 2020) and EthoLoop (Nourizonoz et al., 2020), both of which can perform pose estimation on live video streams at more than 90 FPS on a GPU-enabled computer. The obvious downside, however, is that this approach requires persistent, dedicated access to expensive hardware. This presents a particular hurdle in experiments where multiple video streams must be processed in parallel, such as when monitoring multiple experimental replicates concurrently or using multiple cameras to cover a large behavioral arena.

Here, we present SARTAB (Scalable Automated Real-Time Analysis of Behavior), a modular system for real-time behavior analysis. We then show how it can be used to detect specific courtship behaviors in *P. demasoni* cichlids. Unlike existing GPU-based solutions, our approach uses an Edge TPU (Tensor Processing Unit) peripheral. TPUs, like GPUs, are hardware components that can be used to accelerate neural network inference. Edge TPUs are a subclass of TPUs designed for “edge AI” applications—i.e., lightweight systems that utilize AI (Artificial Intelligence) to process data locally, rather than relying on off-site GPU-enabled servers or the cloud. By pairing an Edge TPU peripheral with the popular Raspberry Pi SBC (Single Board Computer), we achieve AI-assisted real-time behavior analysis in a small, cheap, power-efficient, and self-contained system. We show that despite the computational limitations of our chosen hardware, SARTAB is capable of robust behavior detection under challenging real-world experimental conditions. Although the analysis pipeline we present was built specifically for detecting *P. demasoni* courtship behavior, the intrinsic flexibility of neural networks means that our approach should be adaptable to novel species, environments and behaviors.

## 2 Materials and methods

### 2.1 Tank configuration

**Figure 1:**
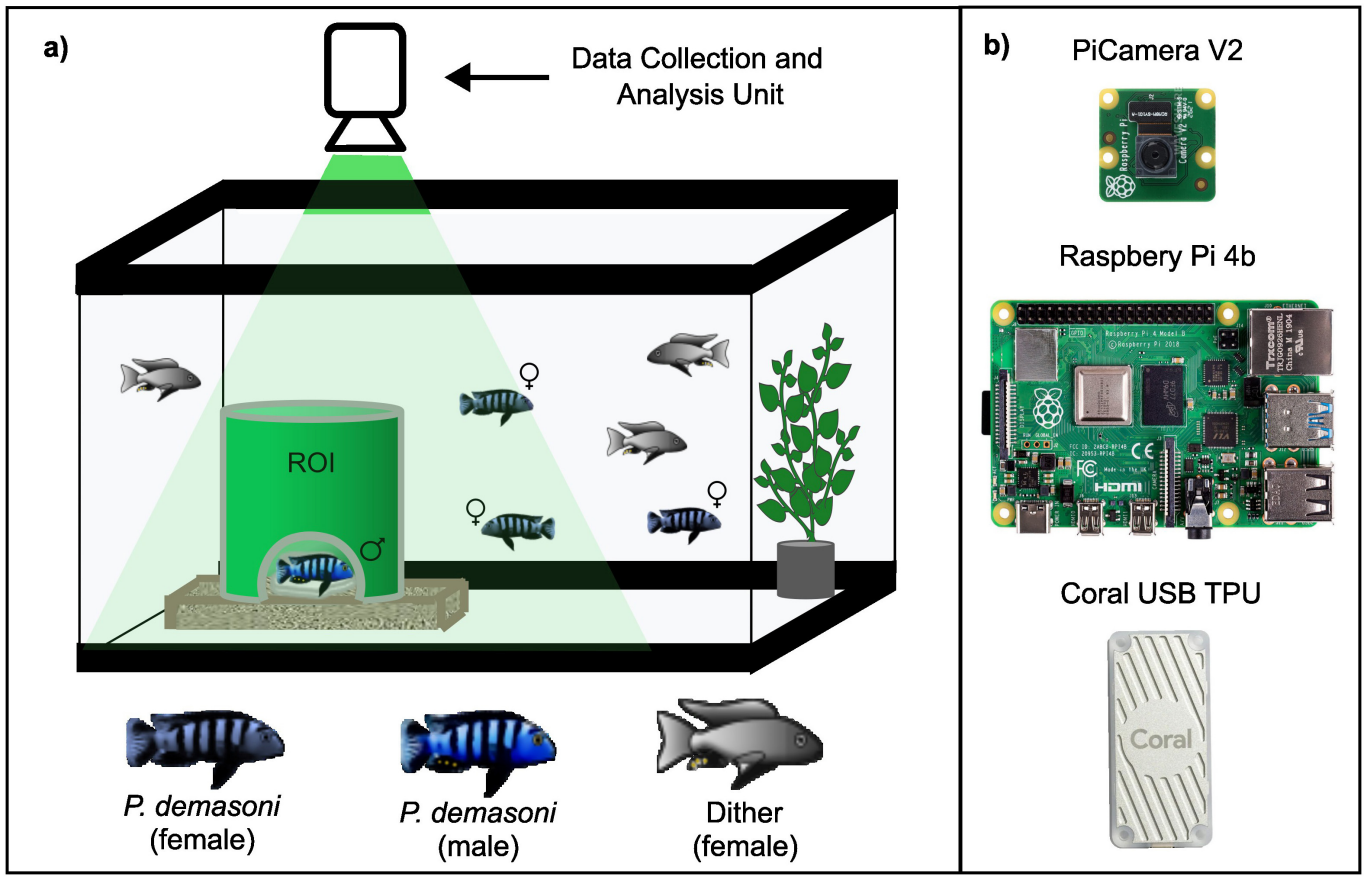
Tank setup and data collection/analysis hardware. **(A)** Each tank is populated with one male and two female *P. demasoni*, as well as three “dither” fish of an unrelated cichlid species. The male will occupy the green pipe and try to entice females inside to mate, making the pipe an important Region Of Interest (ROI). **(B)** Above each tank is a data collection and analysis unit consisting of a Raspberry Pi 4b, a Pi Camera v2, and a Coral USB TPU to accelerate neural network inference.

Cichlids were housed in their home-tank environments, consisting of 50-gallon (~190 L) glass aquariums connected to a centralized filtration system. A tinted film (BDF NA35 from buydecorativefilm.com) was applied to the exterior of the tanks to reduce reflections in the recorded video, and opaque white vinyl shower curtains were used to block visibility between adjacent tanks. To mimic the rocky crevices where *P. demasoni* mate in the wild, we used a 6" length of 6" diameter green PVC pipe (Charlotte Pipe 6" SDR35 Sewer Main Pipe) with a semicircular entrance cut in one side and the top left open for visibility (Figure 1A). The pipe was placed in a shallow acrylic tray (custom built) containing a crushed coral and a rock painted with a white aquarium-safe paint for improved contrast. Each tank was illuminated using a combination of an LED strip light encircling the tank (Lepro 4100058-DW) and a small LED spotlight (Kuange XY0045) pointed downward into the pipe. All lights, including the overhead lights in the room, were connected to timers that turned them on at 7 am and off at 7 pm each day. To eliminate surface ripples (which cause distortion when imaging from above), we submerged a shallow acrylic tray (custom built) just below the water surface. Each tank was stocked with a group of one male and two female sexually mature *P. demasoni*, as well as three “dither” fish – female fish of an unrelated, less aggressive benthic cichlid species (*Mchenga conophoros* X *Copadichromis virginalis* F2 hybrids) that were included to disperse the aggressive tendencies of the focal male.

### 2.2 Recording hardware

Each tank was equipped with a data collection and analysis unit consisting of a single-board computer (Raspberry Pi 4 model B) equipped with a camera (Raspberry Pi Camera version 2.1) and a peripheral TPU (Google Coral USB Accelerator model WA1; Figure 1B). The camera was connected (via a ribbon cable) to the Pi's dedicated camera port, and the TPU (via the included USB cable) to one of the Pi's USB3.0 ports. Each unit also requires a power supply (Raspberry Pi model KSA-15E-051300HU or equivalent), a MicroSD card (SanDisk 256GB Extreme microSDXC or equivalent), and an Ethernet cable (Cat5e or better). At the time of writing, the total MSRP for these parts was $153. Optionally, each unit can be equipped with a screen for ease of interfacing and troubleshooting (e.g., a Raspberry Pi Touch Display) and /or a case for added protection (e.g., a SmartiPi Touch 2). When deploying multiple units, an unmanaged gigabit Ethernet switch can be used to run all network traffic to a single wall port.

### 2.3 Software

Continuous data collection, behavior detection, and system response is executed using custom python code. For the complete code, as well as detailed instructions for installation and use, see: https://github.com/tlancaster6/RBA.

#### 2.3.1 Analysis overview

**Figure 2:**
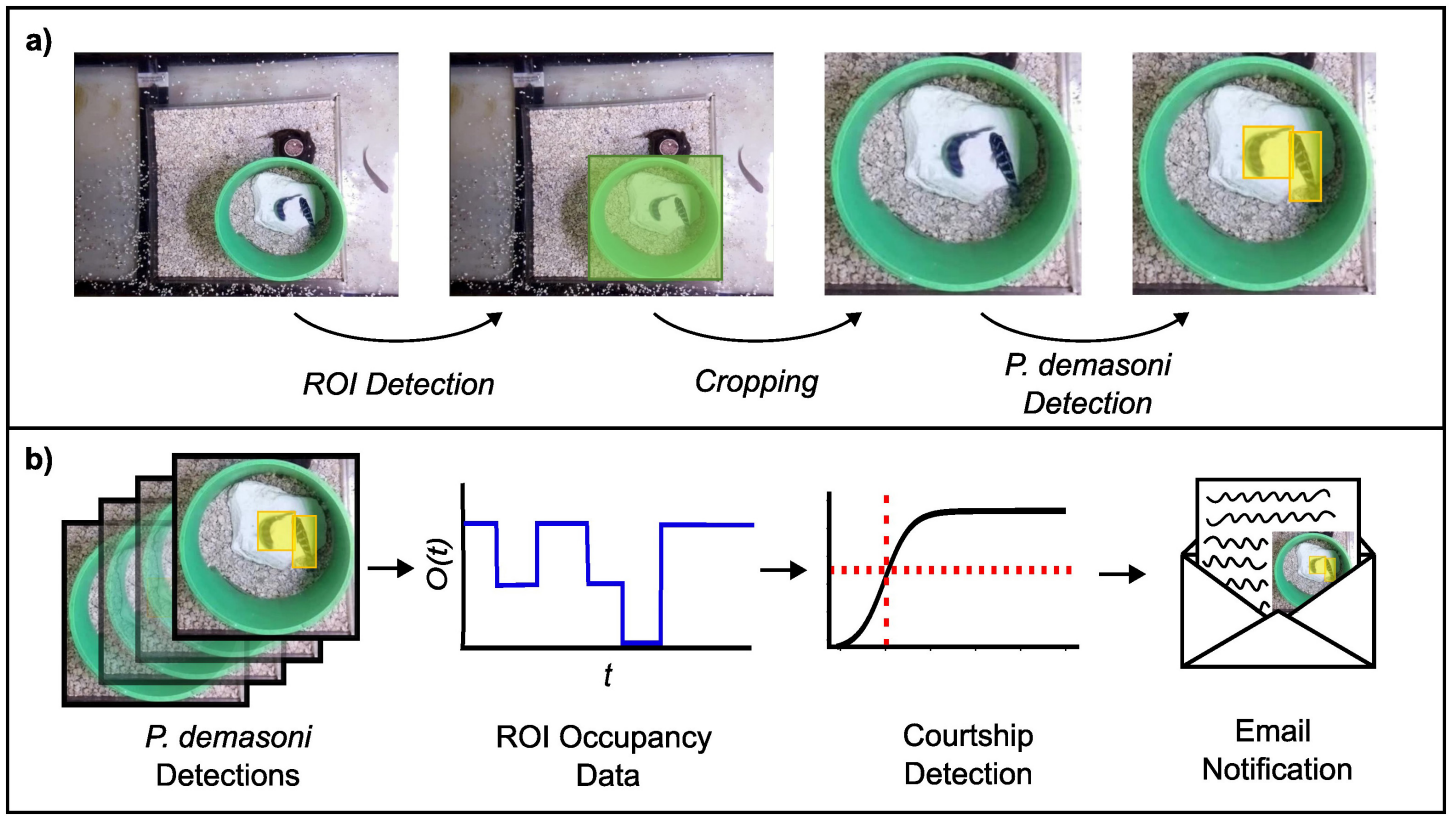
Overview of the SARTAB behavior detection pipeline. **(A)** To determine the number of fish in the ROI (the ROI occupancy) the system uses one object detection network to locate the ROI, and another to locate the *P. demasoni* within an image cropped to the ROI area. **(B)** To infer whether courtship is occurring from these detections, the system periodically calculates the fraction of recent frames where the ROI occupancy was 2 (double occupancy fraction). If the fraction is above a certain threshold (which is pre-computed using a logistic regression classifier) the system generates an email notification which prompts a researcher to collect tissue samples for later multi-omic analysis.

While the behavior of interest (courtship) in our study system is itself complex, it is strongly associated with a comparatively simple (yet sufficient) condition on the animals' locations. Specifically, if we observe exactly two fish within a particular ROI (the green pipe in Figure 2) for more than a few consecutive seconds, we can be relatively confident that courtship is occurring, and vice versa. We leveraged this behavioral insight to design the analysis workflow outlined in Figure 2, which can be divided conceptually into an object detection phase (Figure 2A) and a behavior detection phase (Figure 2B).

The goal of the object detection phase is to determine the number of *P. demasoni* within the Region Of Interest (ROI) in each frame. This is achieved using two object detection networks: one trained to locate the ROI (defined as the rectangle circumscribing the circular mouth of the pipe) in full-frame images, and another trained to locate *P. demasoni* in images cropped to the ROI area. For efficiency, ROI detection is run infrequently (once every 5 min) and the cached results reused to perform the cropping operation. *P. demasoni* detection is run at the highest possible frequency such that the average per-frame processing time is less than the actual time interval between frames (i.e., frames are not generated faster than they can be processed). The number of frames passing through the analysis pipeline per second does not need to match the frame rate of the video being captured for later analysis, but the former must be less than the latter. For a given frame, the number of *P. demasoni* within the ROI (the ROI occupancy) is determined by counting the number of high confidence (*C*>0.5) *P. demasoni* detections.

The goal of the behavior detection phase is to take a series of recent ROI occupancies and determine whether courtship behavior occurred. First, the system calculates ratio of frames where the occupancy was 2 to the total number of frames in the series (double-occupancy fraction). If the double-occupancy fraction is above a set threshold, it concludes that a courtship display is in progress and generates an email notification with a short video clip attached for manual confirmation of courtship. In our system, we ran behavior detection every 30 s using the previous 60 s of occupancy data (resulting in a sliding analysis window with 50% overlap) but these parameters can be easily adjusted via a configuration file.

#### 2.3.2 Object detection model training

For the two object detection tasks (ROI detection and *P. demasoni* detection), we focused on EfficientDet-Lite networks—a family of modified EfficientDet models (Tan et al., 2020) optimized to run on Coral TPUs. We tested both Lite0 and Lite1 size variants, as the larger variants must be pipelined through multiple TPUs to function. We also trained YOLO-V5s models (a recent variant of the popular YOLO architecture (Redmon et al., 2016) for *P. demasoni* and ROI detection. While this model is not compatible with the Coral TPU, it was useful for benchmarking the accuracy of EfficientDet-Lite networks against state of the art models run on traditional GPU-enabled workstations. A summary of the properties of each network variant used in this paper can be found in Table 1.

**Table 1:** Properties of the network architectures used in this paper.

**Architecture**	**Quantized**	***mAP@*[.5:.95] on COCO**	**Model size (MB)**	**Input dimensions (pixels)**
EfficientDet-Lite0	Yes	0.257	4.56	320x320
EfficientDet-Lite1	Yes	0.306	6.08	384x384
YOLO-V5s	No	0.374	14.81	640x640

Values taken from: https://www.kaggle.com/models/tensorflow/efficientdet/tfLite and https://github.com/ultralytics/yolov5.

All ROI detection models were generated using a set of 99 manually-annotated full-frame images (720 x 1280), each with exactly one ROI annotation. All *P. demasoni* detection models were generated using a set of 2,228 manually-annotated images pre-cropped to the ROI area (average resolution 402 x 408) containing an average of 1.05 annotations per image. For both annotation sets, we used a 90:10 train test split. Images were sampled from videos of nine different tanks (each with a unique set of individual fish) to maximize generalizability. For the ROI detection dataset, images were sampled uniformly from full-day videos. For the *P. demasoni* detection dataset, images were generated by first performing a dense uniform sampling of full-day videos, then using the FiftyOne library to bootstrap a smaller dataset of highly unique images.^1^

All models were trained for 50 epochs on a desktop equipped with a NVIDIA RTX4070ti GPU. EfficientDet models were trained using the TensorFlow Lite ModelMaker api, converted to quantized TensorFlow-Lite models using full-integer post-training quantization, and finally compiled for execution on the Edge TPU using the Edge TPU Compiler command-line tool.^2^ The YOLO-V5s model was trained using the official YOLO-V5 software (version 7.0).^3^ All models were initialized with COCO pretrained weights.

#### 2.3.3 Object detection model evaluation

Model performance was primarily evaluated using standard COCO metrics, and reported here in terms of *mAP@*[0.5:0.05:0.95] (shortened to *mAP@*[.5:.95] henceforth). For comparisons between EfficientDet models, COCO metrics were calculated both pre- and post-quantization, and average per-frame inference speed was measured under realistic experimental conditions. One-tailed two-sample t-tests were used to establish the significance and directionality of differences in average inference speed between models. The impact of training set size on *P. demasoni* detection accuracy was quantified by retraining a model with progressively smaller subsets of the training dataset and comparing the *mAP@*[.5:.95] on the original validation set. The relative contribution of dither fish vs. background objects to false positive detections was explored using a modified version of the original validation set with dither fish annotated. One-tailed Mann-Whitney U tests were used to establish the significance and directionality of differences in the confidence score distributions between detections associated with each of the three possible ground truth labels (*P. demasoni, dither, and none*).

#### 2.3.4 Behavior detection model training and evaluation

During operation, our system detects the behavior of interest using a simple threshold on the double-occupancy fraction summary statistic; if the double-occupancy fraction is above this threshold, the system infers that courtship is likely occurring, and vice versa. The threshold value was determined empirically using a binary logistic regression classifier. First, a reference set of 32 1-min clips (16 with courtship, and 16 without) were manually extracted. Next, the double-occupancy fraction each clip was calculated automatically using the object detection approach described above. This data was then used to train a binary logistic regression classifier (SciKit-Learn LogisticRegressionCV model with default parameters and a training fraction of 0.8). Finally, the double-occupancy threshold was set such that *P*(*x*_*cutoff*_) = 0.5. Classification accuracy was calculated for both the training and validation set. To determine whether this approach was robust to sparseness in the occupancy data, inference framerates ranging from 30 fps (i.e., inference performed on and occupancy estimated for every frame of the video) to 1fps (i.e., inference and occupancy estimation only performed on every 30th frame) were simulated and the effects on double-occupancy fraction and classification accuracy explored.

## 3 Results

In this paper we present SARTAB, a system for automatically detecting courtship displays in *Pseudotropheus demasoni*. We developed this system to facilitate the collection of tissue samples containing short-lived behaviorally-relevant neural biomarkers for multi-omic analysis. Previously, our lab has used a similar approach to explore the transcriptomic landscape of courtship in bower-building cichlids (Johnson et al., 2020b; Long et al., 2020; Johnson et al., 2023). In these species, males build elaborate sand structures (bowers) to attract mates. By monitoring the bower construction process, we could easily distinguish between behaving (actively building) and non-behaving males. The cichlid fish *P. demasoni* (the focal species of this study) belongs to the mbuna ecogroup, which inhabits the rocky shorelines of Lake Malawi. Mbuna cichlids do not build bowers; instead, males establish a territory around a protected cave or crevice, then try to entice females into their territory to mate. In the lab, a short length of PVC pipe stands in for the cave. Observing two fish within this pipe is a strong indicator of courtship behavior, and vice versa. To detect courtship behavior in real-time, and thus facilitate neurogenomic analysis, we developed a system that uses object detection to continuously monitor the number of individual *P. demasoni* within the Region Of Interest (ROI) defined by the pipe, then infers whether courtship is occurring from patterns in this data (see Figure 2 and section 1.31 for additional details).

### 3.1 Object detection performance

The first stage of the behavior detection pipeline uses a pair of object detection networks to locate the ROI (pipe), then locate the *P. demasoni* within the ROI. For each of the two detection tasks, we trained and evaluated three models: EfficientDet-Lite0, EfficientDet-Lite1, and YOLO-V5s. Note that because the YOLO-V5s model is incompatible with our TPU-based system, it is excluded from some analyzes.

#### 3.1.1 EfficientDet-Lite accuracies are within 10% from YOLO-V5s accuracies

**Figure 3:**
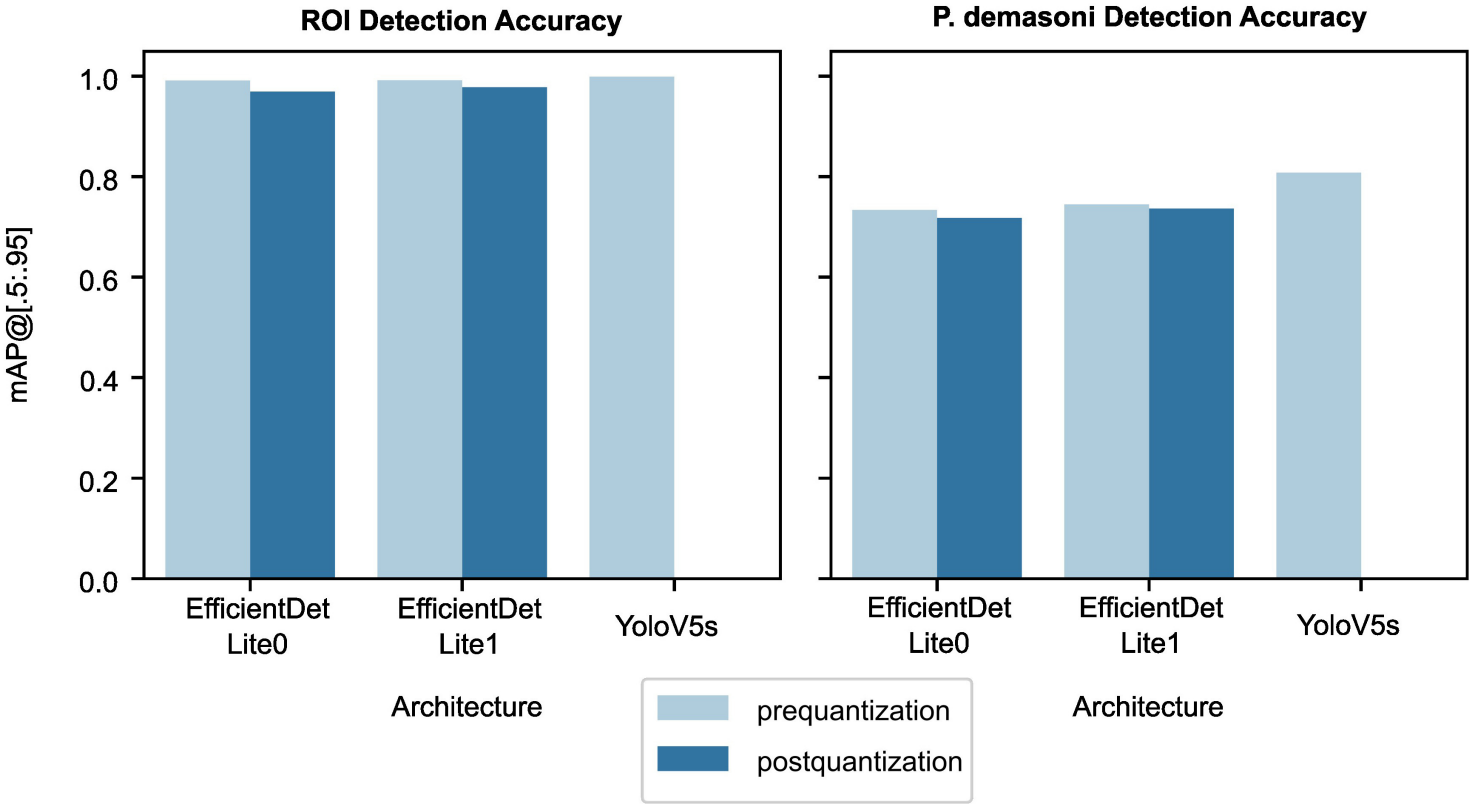
Effect of architecture, detection target, and quantization stage on object detection accuracy. The detection target (ROI vs. *P. demasoni)* has the largest effect on accuracy, as seen from comparing the left and right plots. For both detection tasks, YOLO-V5s achieved the highest accuracy, followed by EfficientDet-Lite1, then EfficientDet-Lite0. Post-quantization accuracies (dark blue) were marginally lower than their respective pre-quantization accuracies (light blue). Note that, for YOLO-V5s models, only pre-quantization accuracy is shown.

The YOLO-V5s model outperforms both EfficientDet-Lite models on both tasks (Figure 3 and Table 2). This is to be expected, as YOLO-V5s is not quantized and uses advanced augmentation techniques during training. The performance difference was most significant between the YOLO-V5s and EfficientDet-Lite0 *P. demasoni* detection networks (*mAP@*[.5:.95] of 0.808 and 0.718, respectively). Considering that the EfficientDet-Lite models can run on an inexpensive TPU, while the YOLO-V5s model requires a GPU (or at the very least a high-power CPU), an accuracy reduction of at most 0.09 seems reasonable.

**Table 2:** *mAP@*[.5 : .95] values from Figure 3.

**Architecture**	**Quantized**	**Detection target**	***mAP@*[.5:.95]**
EfficientDet-Lite0	No	ROI	0.991
EfficientDet-Lite0	Yes	ROI	0.969
EfficientDet-Lite1	No	ROI	0.992
EfficientDet-Lite1	Yes	ROI	0.978
YOLO-V5s	No	ROI	0.999
EfficientDet-Lite0	No	*P. demasoni*	0.734
EfficientDet-Lite0	Yes	*P. demasoni*	0.718
EfficientDet-Lite1	No	*P. demasoni*	0.745
EfficientDet-Lite1	Yes	*P. demasoni*	0.737
YOLO-V5s	No	*P. demasoni*	0.808

#### 3.1.2 Quantization has little effect on performance

For both detection tasks, and for both EfficientDet-Lite variants, quantization appears to reduce accuracy, but only by a small amount (Figure 3 and Table 2). During quantization, a model's internal parameters are converted from float32 to int8 values. This reduces model size 4x and approximately triples inference speed,^4^ but usually at the cost of accuracy. In our case, however, the accuracy difference was marginal, with a maximum observed *mAP@*[.5:.95] loss of 0.022.

#### 3.1.3 EfficientDet-Lite0 models are slightly less accurate but significantly faster than Lite1 models

**Figure 4:**
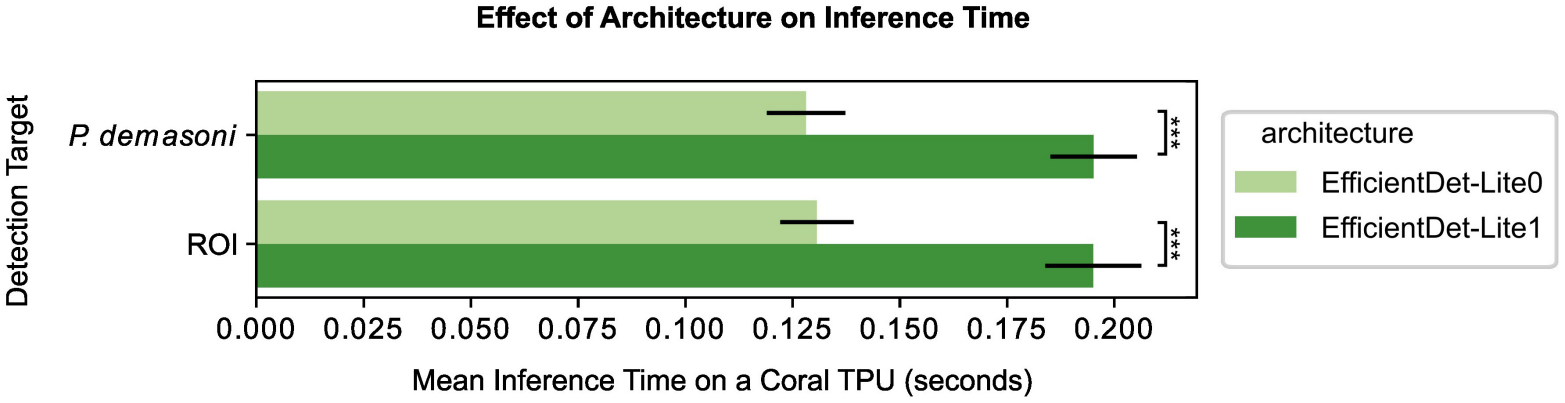
Effect of architecture and detection target on inference speed. For both detection targets (ROI and *P. demasoni*), the EfficientDet-Lite1 architecture (dark green) had a significantly higher mean inference time than the EfficientDet-Lite0 architecture (light green). Note all times refer to inference speed for a quantized model running on a Coral TPU, hence YOLO-V5s is excluded due to incompatibility. ^***^Indicates *p* < 0.001 (see methods).

The EfficientDet-Lite1 architecture achieved slightly higher accuracy than the EfficientDet-Lite0 architecture on both tasks (Figure 3 and Table 2), but was significantly slower (Figure 4). The Lite1 variant, having more parameters and a larger input resolution, is expected to achieve higher accuracy than the Lite0 variant, but at the cost of inference speed. We found that, on average, the *mAP@*[.5:.95] of our Lite1 models was 0.014 higher than our Lite0 models (a 1.6% average relative increase), while the per-image inference time was 0.066 s longer (more than a 50% average relative increase). *T*-tests confirm that the Lite1 average inference time is significantly (*p* < 0.001) higher than the Lite0 average inference time for both detection targets (ROI and *P. demasoni*). We did not perform a statistical comparison of the *mAP@*[.5:.95] results because the observed differences between model variants were so small that, even if technically significant, they would not have influenced our choice of model. Because inference speed is critical for achieving real-time performance, and the observed accuracy difference was marginal, we selected the EfficientDet-Lite0 architecture for both detection tasks.

#### 3.1.4 High accuracy is achievable with relatively few training samples

**Figure 5:**
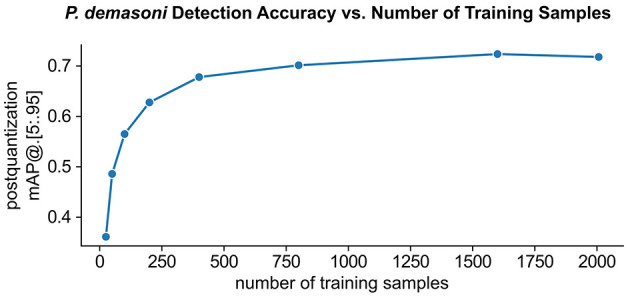
Effect of training set size on *P. demasoni* detection accuracy. The EfficientDet-Lite0 post-quantization *mAP@*[.5 : .95] for *P. demasoni* detection generally increases with the number of training samples, but there is a clear diminishing return on annotation investment.

Model performance generally increases with training set size, but so does the amount of manual annotation work required. While we found that *P. demasoni* detection accuracy generally increased with training set size, we were also able to achieve reasonable accuracies with relatively few annotations (Figure 5). Using the full set of 2007 training images and an EfficientDet-Lite0 model, we achieved a post-quantization *mAP@*[.5:.95] of 0.718 on the validation set (221 images). Using a random subset of just 200 images from the training set, we achieve a *mAP@*[.5:.95] of 0.628 on the same validation set. This suggests that we may have been able to achieve satisfactory results with significantly less annotation effort.

#### 3.1.5 Dither fish have minimal impact on *P. demasoni* detection accuracy

**Figure 6:**
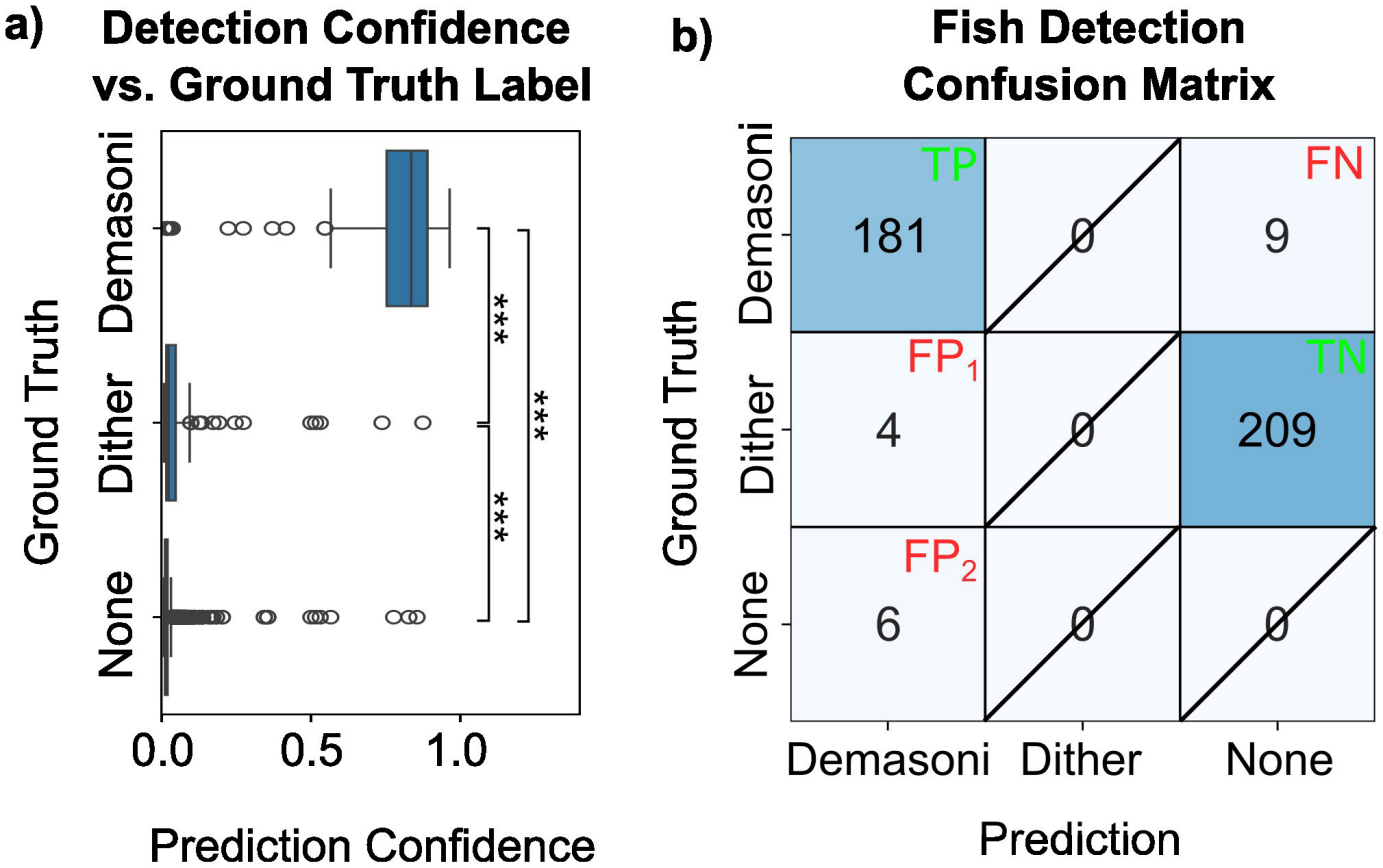
Impact of dither fish on *P. demasoni* detection accuracy. **(A)** Confidence scores for demasoni predictions, separated by the actual ground-truth label. Overall, the model correctly assigns high-confidence scores to correct predictions **(top)**, and low confidences to potential false positives (middle and bottom). Among the false positives predictions, those associated with dither fish **(middle)** were higher confidence than those associated with background objects **(bottom)**. ^*^^*^^*^Indicates *p* < 0.001 (see methods). **(B)** Confusion matrix for a confidence threshold of 0.5 and IOU threshold of 0.6. Note that, in a standard 2-class confusion matrix, the box marked “true negative” (TN) would be considered false negative (FN). However, because dither annotations appear in our ground truth data, but not our training data, the model ignoring the dither fish is in fact the correct behavior, and therefore marked TN. Outcomes that are not possible within our analysis paradigm are marked with a diagonal slash.

Each tank contains three *P. demasoni* (the species of interest) but also three “dither” fish of an unrelated, non-aggressive cichlid. While the *P. demasoni* detection network was trained to ignore the dither fish, we were nevertheless concerned that mis-identification of dither fish as *P. demasoni* might be a major failure mode for our system. Our analysis, however, indicates that the impact is negligible. As shown in Figure 6A, *P. demasoni* true-positives (top) have a significantly higher prediction confidence (*p* < 0.001) than either dither-related (middle) or background-related (bottom) false-positives. As such, even a conservative confidence threshold of 0.5 results in relatively few false-positives (10 total in our validation set), as illustrated in Figure 6B. We also observed that dither-related false positives had statistically significantly higher confidence scores (*p* < 0.001) than background-related false positives, but this has minimal impact on the results as only outliers within these groups exceed the cutoff threshold of 0.5. Of the ten false-positives, four matched closely with the location of a dither fish, while the other six were likely due to background objects. For the remaining 209 dither annotations, the model does not return a prediction (which, since the model is trained to ignore dither fish, is the correct outcome). Of the 191 total *P. demasoni* predictions (left column of the confusion matrix), 181 were in fact *P. demasoni* (i.e., precision = 0.948). Of the 190 *P. demasoni* annotations (top row of the confusion matrix), 181 were correctly detected (i.e., recall=0.953). Based on this, we can conclude (1) that the network performs effectively in detecting *P. demasoni*, as indicated by the high recall score, and (2) that while dither fish may be causing some high-confidence false positives, the high precision score suggests that false positives in general are relatively rare.

### 3.2 Behavior classification performance

In the second stage of the behavior detection pipeline, ROI occupancy data (generated from the object detection phase) is used to infer whether courtship is occurring. This is done by taking a short series of the most recent ROI occupancy measures, determining the fraction of frames in which the ROI occupancy was 2 (double-occupancy fraction) and comparing the result to a pre-computed threshold. If the double-occupancy fraction is above the threshold, the system sends an email notification with a video of the potential behavioral event. After human confirmation of the automated conclusion (via both video review and confirmation of the presence of fertilized eggs), we quickly collect and cryopreserve brain tissue samples for single-nucleus transcriptomic profiling and chromatin accessibility analyzes.

#### 3.2.1 A logistic regression classifier consistently detects courtship behavior

**Figure 7:**
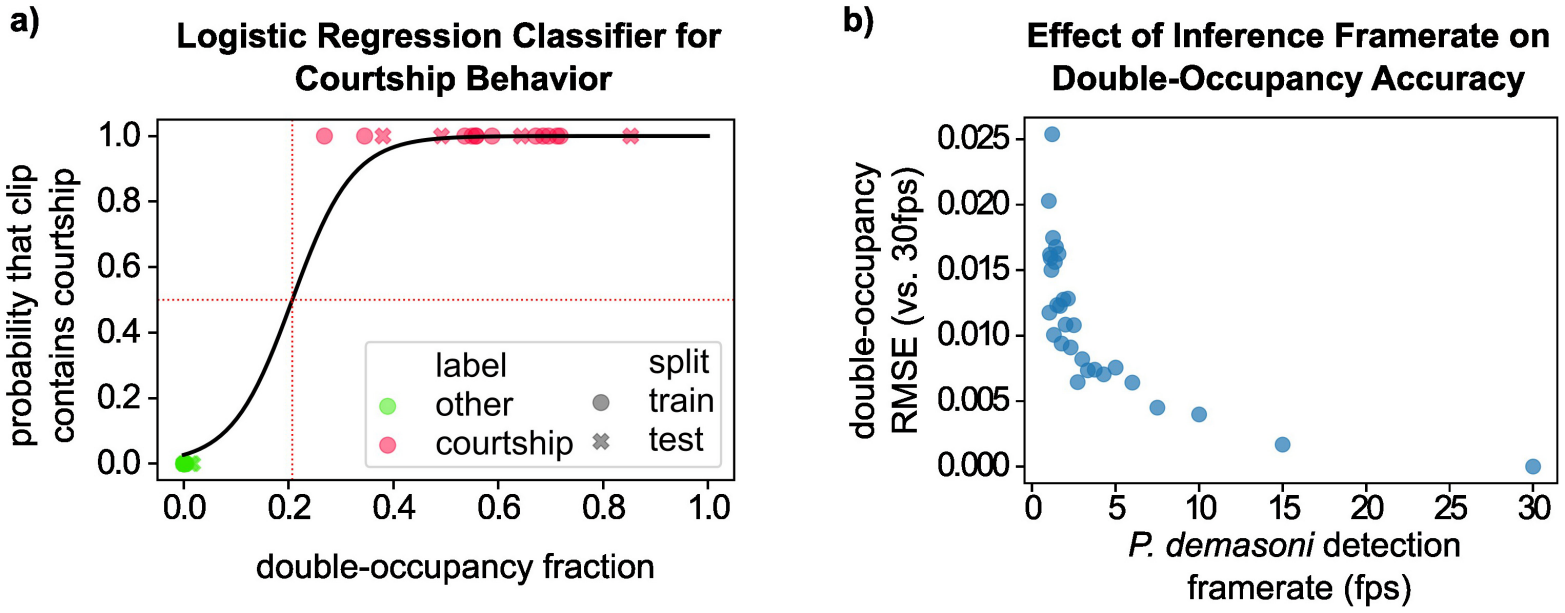
Validation of the logistic regression courtship behavior classifier. **(A)** Visualization of the logistic regression classifier used to identify courtship behavior based on double-occupancy fraction. Note that the classification threshold (vertical dotted red line) correctly separates all courtship clips (pink) and non-courtship clips (green). **(B)** Analysis of the robustness of the double-occupancy metric to variations in the *P. demasoni* detection framerate. While the double-occupancy metric was less accurate at lower framerates, the error was never large enough to cause an incorrect classification in our testing data.

In applying our behavior detection approach to a set of 32 manually-selected video clips (16 with courtship, and 16 without) we found that the double-occupancy fraction separates clearly between the two categories, such that any double-occupancy threshold in the range (0.02, 0.27) correctly classifies all 32 clips. To choose a threshold empirically, and to ensure our methods can be applied to less clear-cut cases, we used a binary logistic regression classifier (Figure 7A). This yields a double-occupancy fraction of 0.207 (vertical red dotted line) as a reasonable threshold for classifying courtship clips, though the classifier could be made more (or less) sensitive by reducing (or increasing) the probability target (horizontal red dotted line).

#### 3.2.2 Inference framerate has minimal impact on behavior classification accuracy

In the previous section, we calculated double-occupancy fraction for each test clip using every available frame (i.e., 1,800 frames per 1-min clip). To achieve this during real-time operation would require a per-frame processing time below 33.3 ms, whereas our EfficientDet-Lite0 inference times ranged from 128 to 131 ms. Our observed inference speeds suggest a theoretical maximum frame rate around 7.5 FPS, but in practice we ran our systems at or below 5 FPS to leave headroom for other processes (e.g. writing the video to disk at a 30 FPS framerate). Based on our analysis, however, we expect similar behavior detection accuracy at 5 FPS as we would see at 30 FPS. To determine this, we first repeated our logistic regression analysis from the previous section, but this time calculated the double-occupancy fraction for each clip using only every 6th frame (equivalent to uniformly sampling 30 FPS video at 5 FPS). The result is visually indistinguishable from Figure 7A, with the classification threshold shifted by < 0.004. Next, we quantified how reducing the framerate affected the double-occupancy metric that underlies the classification (Figure 7B). We assumed that the double-occupancy fraction calculated using every frame (i.e., at 30 FPS) represented our most accurate value for each clip. We then recalculated the double-occupancy fractions using every other frame (15 FPS), every third frame (10 FPS), and so on, all the way up to every 30th frame (1 FPS). For each FPS value, we then calculated the double-occupancy fraction RMSE (compared to 30 FPS) across all 32 clips. At 5 FPS, this corresponds to a double-occupancy fraction RMSE of < 0.008. In general, the RMSE tends to increase (and become increasingly stochastic) as the FPS decreases. But even at very low FPS, the error is still too small to cause any incorrect classifications in our testing data.

## 4 Discussion

Here, we have presented SARTAB, a novel system for real-time detection of specific courtship behaviors in a species of Lake Malawi African cichlid fish. We demonstrate how this system achieves high ROI and animal detection accuracies (*mAP@*[.5:.95] of 0.969 and 0.718, respectively) as well as 100% classification accuracy on a set of 32 manually selected behavioral clips. Our system uses inexpensive consumer-grade hardware, including Raspberry Pi SBCs and Coral Edge TPUs, to achieve CV-assisted behavior analysis. Unlike in most ML-capable behavior analysis workflows, SARTAB centers around modular, self-contained recording and analysis units deployed at the point of data collection. For small projects, this reduces total cost significantly by better matching hardware capabilities to actual computational requirements. For larger projects it has the advantage of simple scalability; units can be added and removed as needed without worrying about bandwidth optimization, GPU multi-instancing, packet collision avoidance, and other such barriers to scaling within traditional or cloud-based computing architectures. In the context of laboratory computational ethology, this scalability is particularly valuable for studying infrequent behaviors under naturalistic conditions, as running numerous experimental replicates in parallel maximizes the overall frequency with which the behavior occurs. This makes our system a valuable tool in behavioral neuroscience for exploring the neuronal repertoire responsible for regulating behavioral plasticity in response to novel and or infrequent stimuli.

While we developed SARTAB for a specific use case, the approach we present here can be adapted to a wide variety of experimental paradigms. With minimal modification, our system could be applied to detect any behavior with a strong location-dependency, but due to the intrinsic flexibility of ML and CV analysis the possibilities are even broader. For example, while we focus on object detection networks in our application, the Google Coral platform is capable of numerous behaviorally relevant CV tasks, including semantic segmentation (Gabdullin and Raskovalov, 2023), pose estimation (Dos Santos Melício et al., 2021), facial expression recognition (Mohammadi et al., 2023), and image classification (Routis et al., 2024). And with the wide range of accessories available for the Raspberry Pi, our “video in, email notification out” design is just one of many possible input-output configurations. Sensors such as microphones, thermometers, accelerometers, RFID readers, and motion sensors can be combined to collect complex multi-modal behavioral data for real-time ML analysis (see Couzin and Heins, 2023 for details on these and other behavioral data modalities). The results can then be used to intelligently control peripherals (e.g., servo motors, LED displays, microfluidic pumps, etc.) to create true closed-loop behavioral experiments (see Nourizonoz et al., 2020; Kane et al., 2020 for examples using GPU-based systems).

While TPU-based systems have many advantages over traditional GPU-based systems, it is important to understand their limitations when designing a computational behavior analysis approach. Notably, Edge TPUs are much less powerful than modern GPUs. This is unsurprising considering the price difference between an Edge TPU system like ours (MSRP $153) and an entry-level desktop with a current-generation Nvidia GPU (MSRP $2000+). In practice, this manifests as increased inference latency and incompatibility with larger, more complex network architectures. In our application, for example, we recorded a minimum per-frame inference time of 128 ms, which was significantly higher than the actual time (33.3 ms) between frame captures. To overcome this, we only processed every 6th frame captured (~5 frames per second) and based our behavioral classification on rolling-window metrics calculated over multiple frames. While the rolling-window width can be reduced to minimize the lag between behavior occurrence and behavior detection (at the cost of reduced resiliency to false positives/negative detections), the inference latency sets a hard lower limit on speed at which behavioral data becomes available to the system. Because we were interested in a behavior that occurs on the scale of seconds to minutes, this limitation was acceptable, and we show that our approach classifies courtship vs. non-courtship clips just as accurately when running at 5 FPS as it would running at 30 FPS. We further believe that this limitation would be acceptable, and therefore that our system would be applicable with minimal modification, to many classes of commonly studied behaviors, such as feeding, nesting, shelter-seeking, and exploration.

If, however, the behavior of interest spans only a few frames, or the system needs to respond to behavioral occurrences near-instantaneously, more powerful hardware will be required to achieve higher inference speed. Similarly, real-time detection of behaviors characterized by fine-scale motion (rather than bulk position) will likely necessitate hardware capable of rapid pose estimation and simultaneous processing of the resulting output (see Kane et al., 2020 for an example). As such, we encourage other researchers to carefully consider their experimental needs, and how those needs may evolve over time, before committing to a hardware platform. We further recommend that, due to the added complexity of real-time analysis, it should be treated as a tool in addition to (rather than a replacement for) bulk offline analysis. That is why we designed our system to not only to detect behavior in real-time, but also to capture and archive behavioral video for more nuanced analysis at a later date.

For applications requiring more powerful hardware there exist a number of TPU-based and GPU-based options. The range of available TPU-based systems is currently limited, but the Coral Dev Board^5^ (MSRP $129.99) is likely better optimized for TPU inference the Raspberry Pi as its operating system was built specifically for TPU support and the TPU itself is integrated at the board level. Alternatively, it is possible to accelerate inference by connecting multiple Coral USB Accelerators (the same TPU peripheral used throughout this manuscript, MSRP $59.99 each) to a single computer,^6^ though this configuration would likely exceed the capabilities of a Raspberry Pi. While we did not test either of these TPU-based hardware solutions, we expect our software would adapt to them easily due to pre-existing TPU compatibility. In scenarios where a TPU-based device is insufficient, but a fully-fledge GPU-enabled workstation or server is excessive, a system from the developer kits from the Nvidia Jetson product line^7^ might provide a reasonable middle-ground. These devices are GPU-based (and therefore capable of running higher-accuracy non-quantized networks) but still achieve a small form factor and reasonable price-point (MSRP $499 for the Jetson Orin Nano Developer Kit). Ultimately, however, GPU-enabled workstations and servers remain the most powerful and flexible solution, and despite their price are likely the best solution for applications requiring maximal accuracy and minimal latency. Adapting our system to run on GPU-enabled devices would require modification of the object detection script (which currently leverages the TPU-specific pycoral API) and likely the data collection script (which currently assumes the input device is a Pi Camera), but due to the modular structure of our code-base should be achievable by any experienced python programmer.

Going forward, broad commercial interest in Edge-AI will continue to drive the development of inexpensive, compact devices with impressive ML capabilities (Singh and Gill, 2023). As we have shown in this paper, these devices have significant potential applications within the fields of computational ethology and behavioral neuroscience, and are particularly well-suited to building systems for real-time behavior analysis. In a laboratory setting, they can form the basis of cheap and highly flexible systems for facilitating large-scale, long-term studies under challenging naturalistic conditions. Beyond the lab, these devices have rich potential applications in field settings, where their low power-draw and small form-factor would make them ideal for intelligent animal and environmental monitoring systems. Finally, the same technologies found in devices like the Coral are increasingly finding their way into smartphones and other consumer technologies, presenting exciting possibilities for ML-driven citizen science and STEM education.

## Data Availability

The raw data supporting the conclusions of this article will be made available by the authors, without undue reservation.

## Footnotes

^1^ https://github.com/voxel51/fiftyone-examples/blob/master/examples/image_uniqueness.ipynb

^2^ https://github.com/google-coral/tutorials/blob/master/retrain_efficientdet_model_maker_tf2.ipynb

^3^ https://zenodo.org/records/7347926

^4^ https://www.tensorflow.org/lite/performance/post_training_quantization

^5^ https://coral.ai/products/dev-board

^6^ https://coral.ai/docs/edgetpu/pipeline/

^7^ https://www.nvidia.com/en-us/autonomous-machines/embedded-systems/
